# The influence of carboxymethyl cellulose and hydroxypropyl methylcellulose on physicochemical, texture, and sensory characteristics of gluten‐free pancake

**DOI:** 10.1002/fsn3.3844

**Published:** 2023-11-20

**Authors:** Maryam Maghsoud, Ali Heshmati, Mehdi Taheri, Aryou Emamifar, Fatemeh Esfarjani

**Affiliations:** ^1^ Department of Nutrition and Food Hygiene, School of Medicine, Nutrition Health Research Center Hamadan University of Medical Sciences Hamadan Iran; ^2^ Department of Food Science and Technology, College of Food Industry Bu‐ Ali Sina University Hamedan Iran; ^3^ Research Department of Food and Nutrition Policy and Planning, Faculty of Nutrition Sciences and Food Technology, National Nutrition & Food Technology Research Institute (NNFTRI) Shahid Beheshti University of Medical Sciences Tehran Iran

**Keywords:** celiac, gluten‐free products, metabolic disease, pancake, texture analyzer

## Abstract

In this study, gluten‐free pancakes were prepared using rice flour and potato starch at a ratio of 50:50. Due to a lack of gluten networks in these ingredients, the hydrocolloid gums including carboxymethyl cellulose (CMC) at 0.5%, 1%, and 1.5% and hydroxypropyl methylcellulose (HPMC) at 1%, 2%, and 3% were added to improve the quality of the final products. The effects of these hydrocolloid gums on the physicochemical, textural, and sensory properties of the gluten‐free pancakes were evaluated. Pancakes prepared with wheat flour were used as a control sample. The results showed that the addition of both gums decreased the hardness and chewiness of the gluten‐free pancakes while increasing the springiness and their moisture content. Increasing the concentration of the gums resulted in an L* value (lightness) reduction, which produced a darker crust on the pancakes. Moreover, the gluten‐free pancakes containing CMC and HPMC had higher specific volumes than the gluten‐free samples made without CMC and HPMC. From a sensory point of view, the samples containing 2%, 3% HPMC and 1% CMC received the highest overall acceptance score. Thus, CMC and HPMC can be used as improvers in gluten‐free pancakes.

## Graphical abstract

The influence of carboxymethyl cellulose (CMC) and hydroxypropyl methylcellulose (HPMC) on properties of gluten‐free pancakes was investigated. The addition of CMC and HPMC decreased the hardness and chewiness of the gluten‐free pancakes. Increasing the concentration of CMC and HPMC resulted in an L* value (lightness) reduction and specific volume increment of pancakes. The gluten‐free pancakes containing 3% HPMC had the highest overall acceptance score.

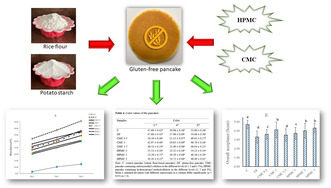

## 1 INTRODUCTION

Celiac disease is a chronic digestive disease that causes sensitivity to the gluten found in wheat, rye, and barley (Sollid, 2002). In celiac patients, intestinal villi are destroyed, and nutrient absorption in the intestine is reduced. The prevalence of celiac disease in Iran and several other countries is reported to be approximately 1% of the population (Shahbazkhani et al., 2003). Celiac disease can occur at any age and can present with various symptoms, including diarrhea, bloating, abdominal pain, constipation, weight loss, anemia, neurological disturbances, and osteoporosis (Fasano & Catassi, 2012; Rubio‐Tapia & Murray, 2010).

Treatment for celiac disease involves following a strict gluten‐free diet. Gluten‐free economical alternatives to wheat, such as corn, rice, starch, and potato flour, are widely used for the production of gluten‐free products (Houben et al., 2012). Starches are effective in retaining moisture and creating the desired color and volume (Anton & Artfield, 2008). Potato flour has particularly favorable characteristics for use in gluten‐free products due to its safe compounds, high water‐binding capacity, and swelling power (Mezaize et al., 2009; Roman et al., 2019; Sivaramakrishnan et al., 2004). Similarly, rice flour is a safe ingredient for gluten‐free food production due to its low‐fat, sodium, and calorie content and high fiber level, and anti‐inflammatory properties (Kiprushkina et al., 2020; More et al., 2013).

Several studies have found that the absence of a gluten network in gluten‐free ingredients results in the final product having weaker textural properties and a shorter shelf life than those using wheat‐based ingredients (Houben et al., 2012; Zoghi et al., 2021). Gluten networks play a significant role in the quality of bakery products and help to retain moisture and improve taste (Biesiekierski, 2017). To improve the rheological, viscoelastic, and organoleptic properties of gluten‐free products, additives, such as emulsifiers, enzymes, or hydrocolloids, are used (Cappelli et al., 2020).

Hydrocolloids are believed to help delay starch retrogradation, maintain moisture, and improve the textural and sensorial properties of gluten‐free products, thus increasing consumer acceptance (Salehi, 2020). Hydrocolloids swell to form a gel that helps retain gas bubbles within the dough, creating a spongy structure and increasing the dough's water‐binding capacity. In addition, hydrocolloids delay gluten‐free products from going stale. As a result, gluten‐free products containing hydrocolloids seem fresher than those without them (Anton & Artfield, 2008).

Various hydrocolloids are used in the production of gluten‐free products (Roman et al., 2019). Among these, carboxymethyl cellulose (CMC) and hydroxypropyl methylcellulose (HPMC) are considered particularly suitable for use in the production of gluten‐free food. The addition of CMC to rice flour has been found to improve the specific volume and quality of the crust properties of gluten‐free bread (Franco et al., 2020). In gluten‐free bread made with corn flour, rice flour, and potato starch, HPMC was found to have a greater impact than xanthan and CMC on the reduction of hardness and increase in volume (Mezaize et al., 2009). In addition, a mixture of CMC (0.8%) and HPMC (3.3%) improved the quality of gluten‐free bread made with rice flour (Cato et al., 2002). In another study, the use of CMC and HPMC gum was reported to enhance the textural properties of gluten‐free bread made using rice flour (Demirkesen et al., 2014).

Many types of gluten‐free products are manufactured, including breads, pancakes, cakes, cupcakes, doughnuts, and muffins. A pancake is a flat cake, often thin and round, made from a starch‐based batter that may contain eggs or milk. It is cooked on a hot surface, such as a griddle or frying pan. Pancakes can be served at any time of the day with a variety of toppings or fillings, and they are a popular breakfast option among children.

Several studies have considered gluten‐free pancake production. For example, Shih et al. (2006) studied the physicochemical properties of gluten‐free pancakes made with rice flour and various percentages of sweet potato starch. They found that the textural properties (hardness, cohesiveness, and chewiness) of the gluten‐free pancakes were comparable to those of wheat flour (control) samples (Shih et al., 2006). Kiprushkina et al. (2020) investigated the production of gluten‐free pancakes with a high nutritional value made with rice and corn flour. They added xanthan gum to the formulation of the gluten‐free pancakes to increase the elasticity of the final products and concluded that the viscosity of the gluten‐free dough was lower than that of the control samples (sample made by wheat flour). In addition, samples with a rice‐to‐corn ratio of 85:15 produced the most positive effect in terms of increasing the sensory scores (Kiprushkina et al., 2020). Similarly, Phongnarisorn et al. (2023) evaluated the effect of xanthan gum on the physical, sensory, and chemical properties of gluten‐free pancakes and waffles made with riceberry flour. Their results indicated that xanthan gum addition (0.25%–1%) decreased the hardness, chewiness, and gumminess of the final product (Phongnarisorn et al., 2023).

Improving the sensory and textural properties of gluten‐free products that lack gluten networks is considered an important issue. To the best of our knowledge, there are several studies about the effect of HPMC and CMC gums on the quality improvements of different gluten‐free products (Cato et al., 2002; Demirkesen et al., 2014; Franco et al., 2020; Mezaize et al., 2009). However, there is no published report about the effect of using HPMC and CMC on the quality of gluten‐free pancakes based on rice flour and potato starch. Thus, this study was performed to add to our knowledge about the effects of HPMC and CMC gums on the sensory, physicochemical, and textural properties of gluten‐free pancakes.

## 2 MATERIALS AND METHODS

### 2.1 Materials

Rice flour was obtained from Golha Company (Tehran, Iran); potato starch was purchased from Alvand Company (Hamadan, Iran); low‐fat pasteurized milk (1.5%) was bought from Pegah Company (Hamadan, Iran); salt, sugar, sunflower oil, fresh eggs, gluten‐free vanilla, and baking powder (Dr. Oetker) were purchased from local market (Hamadan, Iran); and HPMC and CMC hydrocolloids were purchased from Sigma‐Aldrich (St. Louis, MO, USA).

### 2.2 Pancake preparation

**FIGURE 1:**
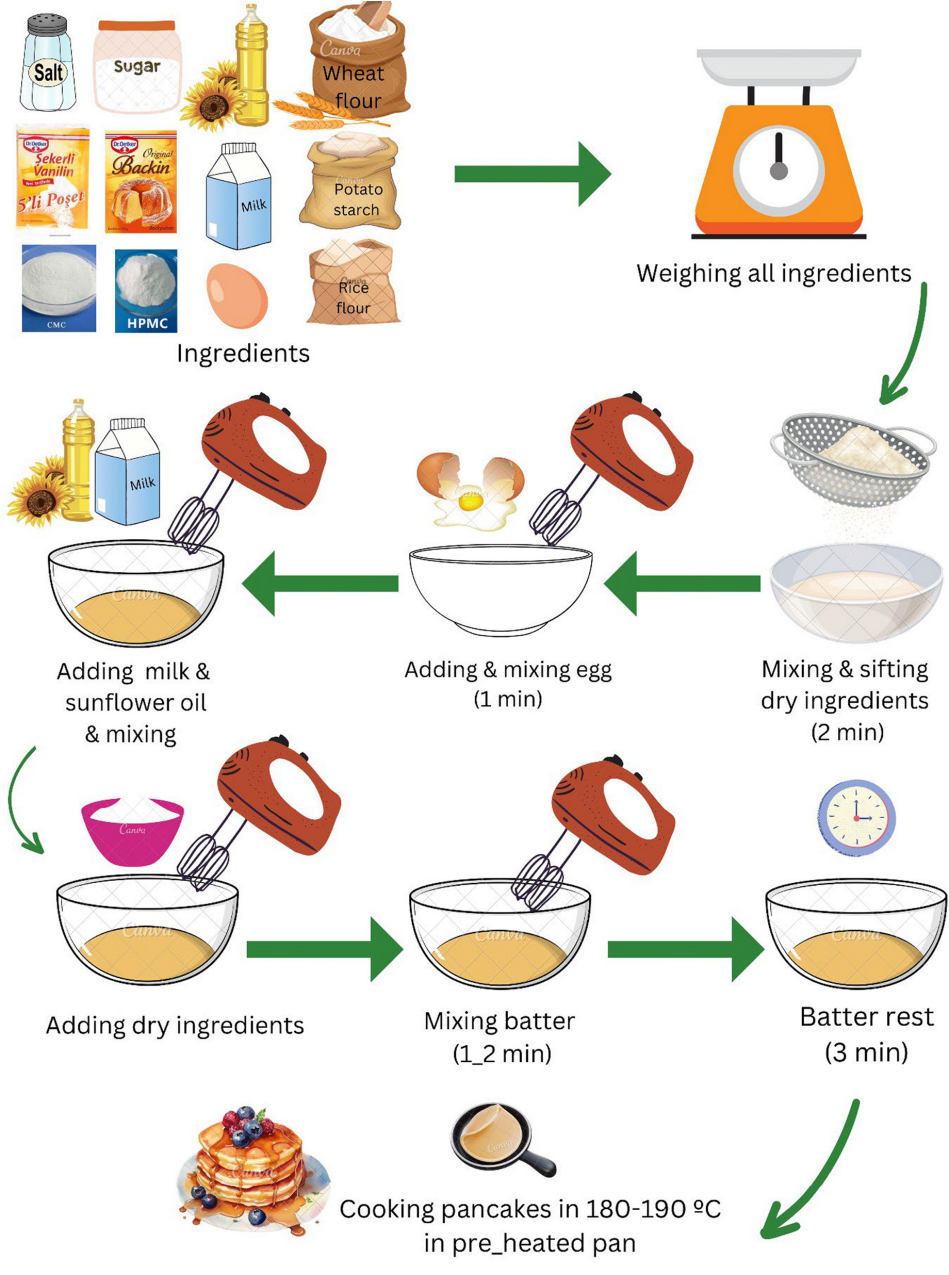
*Infographic im*age of the pancake preparation. Canva application was used to create infographics.

The formulation of the different types of pancakes made in this study is shown in Table 1. All steps of pancake preparation are explained in Figure 1. The gluten‐free pancake was prepared using the following formula: 0.2 g salt, 20 g sugar, 4 g baking powder, 0.5 g vanilla powder, 50 g rice flour, 50 g potato starch, 100 g milk, 6 g vegetable oil, and 57 g whole egg. For gluten‐free pancakes containing gum, other ingredients such as HPMC at 3 levels of 1%, 2%, and 3%, and CMC at levels of 0.5%, 1%, and 1.5% were used. For the control sample (sample containing wheat flour), instead of rice flour and potato starch, wheat flour was used. For the preparation of the pancakes, all ingredients were weighed. Then, the dry ingredients were sifted. The whole egg was mixed with a high‐speed electric mixer (Tefal, model HT 400B30, France) for 2 min in an empty bowl, and the liquid ingredients (milk and vegetable oil) were added and mixed. The dry ingredients were added, and the batter was mixed for 2 min. In final, the pancake batter stayed for 3 min at the ambient temperature. For the cooking of the pancakes, the pan with a flat surface was preheated, and 20–25 g of pancake batter was transferred into the pan at a temperature of 180–190°C for about 2 min until the bubbles appeared on the surface of the pancakes, and then the other side of the pancakes heated for about other 1 min.

**TABLE 1:** Pancake formulations.

Ingredients (g)	Samples							
	C	GF	CMC 0.5	CMC 1	CMC 1.5	HPMC 1	HPMC 2	HPMC 3
Wheat flour	100	–	–	–	–	–	–	–
Rice flour	–	50	50	50	50	50	50	50
Potato starch	–	50	50	50	50	50	50	50
Egg	57	57	57	57	57	57	57	57
Milk	100	100	100	100	100	100	100	100
Sugar	20	20	20	20	20	20	20	20
Salt	0.2	0.2	0.2	0.2	0.2	0.2	0.2	0.2
Sunflower oil	6	6	6	6	6	6	6	6
Baking powder	4	4	4	4	4	4	4	4
Vanilla powder	0.5	0.5	0.5	0.5	0.5	0.5	0.5	0.5
CMC	–	–	0.5	1	1.5	0	0	0
HPMC	–	–	0	0	0	1	2	3

Abbreviations: C, control pancake (wheat flour‐based pancake); CMC, pancake containing carboxymethyl cellulose in the different levels (0.5%, 1%, and 1.5%); GF, gluten‐free pancake; HPMC, pancake containing hydroxypropyl methylcellulose in the different levels (1%, 2%, and 3%).

### 2.3 Physicochemical properties of pancakes

Moisture, protein, and fat were determined according to approved methods of the American Association of Cereal Chemists, No 44‐15A, 46‐10, and 30‐10, respectively (AACC, 2000). Total carbohydrates were determined by Lane Eynon method (Shoaei et al., 2022).

### 2.4 Viscosity of batter

The viscosity of pancake batter was determined using a Brookfield viscometer (DV2T, USA). The pancake batter was transferred to a 250 mL beaker and leveled up to the brim. The spindle speed was set to 150 rpm, and spindle No. 6 was used for all experiments except the gluten‐free sample because of the low viscosity of this sample. The viscosity of the batter was measured after 3 min resting at the temperature of 23 ± 2°C. Finally, the peak viscosity of the pancake batter was reported.

### 2.5 Specific volume of pancake

For the measurement of the specific volume of the pancake, the rapeseed displacement method was used (Mir et al., 2015).

### 2.6 Color analysis

The crust color of the pancakes was evaluated using Image J software (version 1.53a, National Institutes of Health, Bethesda, Md.). The L* scale ranges from 0 (black) to 100 (white): the b* scale ranges from a negative (blue) to a positive value (yellow), while the a* scale extends from a negative value (green hue) to a positive value (red hue). Color changes were also evaluated as the total color difference (Δ*E*) according to the previous study (Mir et al., 2015). Pancake samples were tested about 20 min after completely cooling.

### 2.7 Scanning electron microscopy (SEM)

The microstructure of the pancakes (one day after production) was determined using the Scanning Electron Microscope (SEM, JSM‐840A, JEOL, Japan). The pancake samples were cut into pieces with a size of 5 mm × 5 mm × 5 mm, freeze‐dried for 24 h with a freeze dryer (OPERON, South Korea), and kept in a vacuum‐sealed wrap until measurement. Each pancake sample was placed in a holder, coated with gold, and finally each sample was transferred to microscope for SEM experiment, where it was observed at 15–20 KV with a magnification of 50 and 500.

### 2.8 Texture profile analysis

For texture analysis, a Texture Analyzer (SANTAM, STM5, Tehran, Iran) was used. The parameters of the device were set as follows: Mode: the double‐bite compression; prob type: a flat (2.5 cm); total cycles: 2 steps; test speed: 100 mm/min; target value: 50% deformation; trigger point: 5 g; and the recovery time: 0 s. The texture properties of all samples were determined 20 min after production (0 day), 1 and 2 days after storage in the refrigerator. When the temperature of samples reached ambient temperature, they were examined. Hardness, cohesiveness, springiness, chewiness, and adhesiveness were evaluated from the TPA graphic method similar to the previous study (Gómez et al., 2007).

### 2.9 Sensory evaluation

The sensory properties (color, flavor, texture, taste, and overall acceptance) of the pancakes were evaluated 2 h after production on the first day according to the hedonic scale of five points (1 = dislike extremely, 2 = do not like it, 3 = neither like nor dislike, 4 = like it, and 5 = like extremely). Trained panelists include 10 master's degrees of food industry students of Bu Ali Sina University of Hamadan, Iran (in the age range of 25–30 years old) who were used for sensory testing. For the training of panelists, familiarization sessions were held. They taste and rate different products and compare their ratings with those of other panelists. In addition, the correct and consistent method of tasting, such as rinsing their mouth, and taking small bites was trained. A number code for each sample were considered. Then, the samples were put into the sterile plate with a sterile fork and knife and then given to panelists in their code‐randomized order. Panelists rinsed their mouths after testing each sample. Means sensory scores were used in the analysis (Taghdir et al., 2017).

### 2.10 Statistical analysis

All the experiments were performed in triplicate using SPSS 22:0 Advanced Statistics (IBM; Armonk, NY, USA). The mean ± standard deviation of each experiment was reported. The one‐way ANOVA and Duncan's multiple range test was used to determine significant difference at the level of 95%.

## 3 RESULTS AND DISCUSSION

### 3.1 Physicochemical properties of pancakes

The physicochemical properties of the pancake samples are shown in Table 2. There is no significant difference between the fat and total carbohydrate content of the various samples although moisture content differed among samples. The highest (44.32%) and lowest (37.96%) moisture contents were found in the control (wheat flour) and gluten‐free pancake sample (without gum), respectively. The results revealed that the highest protein content (8.43%) was related to the control sample (wheat flour) due to the presence of gluten (Mir et al., 2015). Also, there was no significant difference in the protein content of gluten‐free pancake samples (*p* < .05).

**TABLE 2:** Physicochemical properties of the pancakes.

Samples	Moisture content (%)	Protein content (%)	Fat content (%)	Total carbohydrate (%)
C	44.32 ± 3.54^a^	8.43 ± 0.67^a^	4.42 ± 0.35	40.26 ± 0.67
GF	37.96 ± 3.19^f^	6.79 ± 0.51^b^	4.50 ± 0.52	40.41 ± 0.71
CMC 0.5	39.55 ± 3.22^e^	6.44 ± 0.55^b^	4.02 ± 0.35	40.32 ± 0.65
CMC 1	41.01 ± 3.29^c^	6.45 ± 0.54^b^	4.64 ± 0.34	40.98 ± 0.60
CMC 1.5	41.77 ± 3.25^bc^	6.36 ± 0.49^b^	4.42 ± 0.38	40.19 ± 0.78
HPMC 1	40.41 ± 3.23^d^	6.40 ± 0.57^b^	4.31 ± 0.42	40.22 ± 0.82
HPMC 2	41.23 ± 3.29^c^	6.34 ± 0.50^b^	4.20 ± 0.39	40.27 ± 0.63
HPMC 3	42.60 ± 3.40^b^	5.96 ± 0.47^b^	4.10 ± 0.31	40.32 ± 0.61

*Note*: Mean ± standard deviation with different superscripts in a column differ significantly (*p* < .05) (*n* = 3).

Abbreviations: C, control pancake (wheat flour‐based pancake); CMC, pancake containing carboxymethyl cellulose in the different levels (0.5%, 1%, and 1.5%); GF, gluten‐free pancake; HPMC, pancake containing hydroxypropyl methylcellulose in the different levels (1%, 2%, and 3%).

Moisture content plays a significant role in the textural properties of bakery products. CMC and HPMC retained more moisture in gluten‐free pancakes. With increasing the concentration of the HPMC and CMC, the moisture content was increased although HPMC had more impact than CMC on moisture retention. The finding regarding higher moisture in greater levels of gum was similar to the previous study. The higher moisture content for gluten‐free doughnuts containing a high concentration of HPMC, CMC, and xanthan was reported in the previous study (Siriwongwilaichat & Kongpanichtrakul, 2021). In addition, the water absorption ability of hydrocolloids was reported in the order, HPMC > CMC > apple pectin > xanthan gums in (Liu et al., 2018) study. In other products such as gluten‐free noodles, high moisture content was reported for samples containing HPMC and CMC (Sutheeves et al., 2020). In contrast, the lower moisture content for gluten‐free crackers containing high levels of CMC (2%) compared to samples containing CMC (1.5%) was found (Nammakuna et al., 2016).

In other studies, the protein value among samples with or without gum had no significant difference. For example, the protein content of chapatti bread without gums, containing 0.5% HPMC or 0.5% CMC, was reported as 11.68, 11.74, and 11.71% respectively (Ahmed et al., 2013). Also, non‐significant (*p* < .05) changes were reported for protein content among the cupcake samples prepared with different concentrations of CMC (Abdelnaby et al., 2021).

### 3.2 Viscosity of pancakes batter

The results of different viscosities of pancake batter are shown in Table 3. Based on the data, the gluten‐free batter had the lowest viscosity (482.67 cP) due to the lack of gluten network. Results showed that by increasing the CMC and HPMC concentration, the viscosity of the pancake batter increased significantly (*p* < .05), particularly in the sample containing 1.5% CMC (12123.35 cP). Also, the lower viscosity of gluten‐free dough was reported in the previous study (Kiprushkina et al., 2020). Generally, hydrocolloids could dramatically affect the flow behavior of foodstuffs even at low concentrations. Also, the higher concentration of HPMC (1.7%) and lower concentration of CMC (0.4%) caused better quality parameters than other gum in bread based on wheat and rice flour (Anton & Artfield, 2008). It was reported the addition of different hydrocolloids to eggless cake based on wheat flour induced greater viscosity of batter compared to the control (without gums) sample (Ashwini et al., 2009). Also, the addition of different gums such as xanthan, fenugreek, flaxseed, and okra except arabic gum increased the peak viscosity of samples compared to control samples (Shahzad et al., 2020). However, with the addition of acacia and apricot gum to gluten‐free cookies, the peak viscosity of rice‐chickpea composite flour was reduced (Hamdani et al., 2020).

**TABLE 3:** Viscosity and specific volume of the pancakes.

Samples	Viscosity (cP)	Specific volume (cm^3^/g)
C	5108.00 ± 3.49^a^	33.07 ± 0.59^a^
GF	482.67 ± 3.96^g^	14.43 ± 0.13^e^
CMC 0.5	1716.17 ± 5.42^f^	19.71 ± 0.86^d^
CMC 1	5430.00 ± 4.71^d^	27.08 ± 0.80^b^
CMC 1.5	12123.35 ± 5.74^b^	27.04 ± 0.46^b^
HPMC 1	2325.00 ± 3.07^f^	23.32 ± 0.35^c^
HPMC 2	4959.50 ± 5.36^e^	28.44 ± 0.45^b^
HPMC 3	9322.50 ± 3.54^c^	31.31 ± 0.36^a^

*Note*: Mean ± standard deviation with different superscripts in a column differ significantly (*p* < .05) (*n* = 3).

Abbreviations: C, control pancake (wheat flour‐based pancake); CMC, pancake containing carboxymethyl cellulose in the different levels (0.5%, 1%, and 1.5%); GF, gluten‐free pancake; HPMC, pancake containing hydroxypropyl methylcellulose in the different levels (1%, 2%, and 3%).

### 3.3 Specific volume of pancake

Specific volume analysis of different pancake formulations is shown in Table 3. Among different external characteristics of bakery products, specific volume is considered the most important visual parameter for the choices of consumers (Liu et al., 2018). Gluten‐free samples without gums had the lowest specific volume (14.43 cm^3^/g), while control samples had the highest specific volume (33.07 cm^3^/g). The addition of HPMC and CMC gum in gluten‐free pancakes resulted in the increment of specific volume compared to gluten‐free samples without gum. However, despite the high viscosity of the sample containing 1.5% CMC, no increment was seen for the specific volume of this sample. In line with our finding, the highest specific volume was reported for control (wheat flour) doughnuts compared to gluten‐free soy doughnuts (Kim et al., 2015). The positive effect of CMC and HPMC gums in the improvement of the volume has been previously reported (Lazaridou et al., 2007). Also, the larger specific volume for gluten‐free potato steamed bread which contains HPMC among different gums has been reported. It seems that HPMC and CMC gums interacted with the protein or starch of the potato, rice, and corn and caused more stable dough and specific volume increment (Liu et al., 2018). In addition, the influence of gums on the final product volume could be due to increase in batter viscosity that slows down the rate of gas diffusion and allows its retention in the initial stages of baking (Ashwini et al., 2009). However, Numfon (2017) found that the addition of 1% xanthan improved the specific volume of bread, while the addition of 2% xanthan caused a drop in the specific volume of bread. A similar trend was observed in Phongnarisorn et al. (2023) study, about the addition of a higher concentration of xanthan gum (2%) in gluten‐free pancakes. It was stated that, due to the higher viscosity and lower flow rate of this sample, the heaviest pancakes with the smallest diameter were produced.

Also, opposite results regarding the impact of gums on the specific volume were reported. For example, Sabanis and Tzia (2011) reported a lower specific volume for bread samples containing xanthan (Sabanis & Tzia, 2011).

### 3.4 Color analysis

**FIGURE 2:**
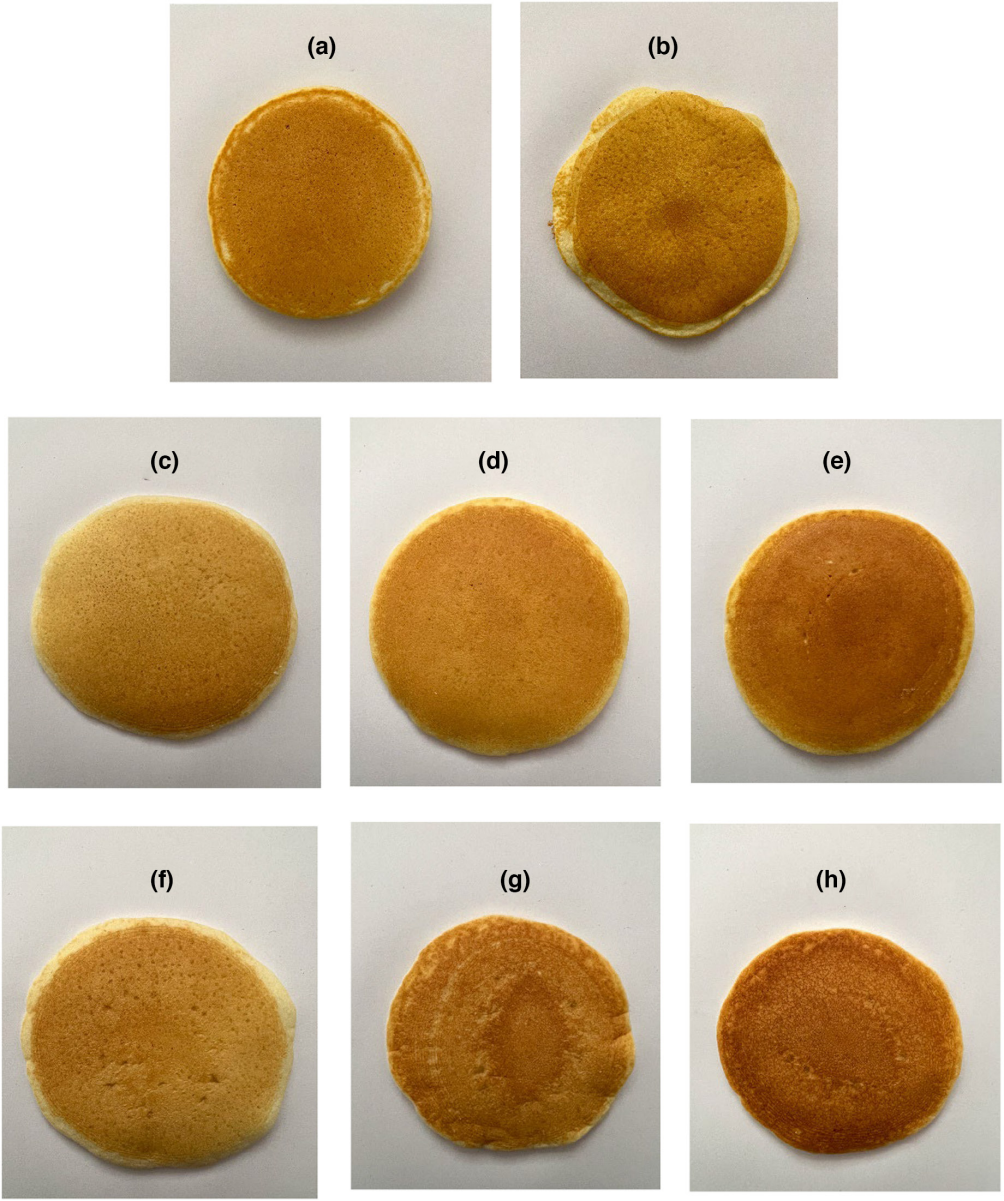
Pancake pictures with different formulations. (a) wheat flour‐based sample (control); (b) gluten‐free sample; (c) gluten‐free sample containing 1% HPMC; (d) gluten‐free sample containing 2% HPMC; (e) gluten‐free sample containing 3% HPMC; (f) gluten‐free sample containing 0.5% CMC; (g) gluten‐free sample containing 1% CMC; (h) gluten‐free sample containing 1.5% CMC.

The pancake pictures are shown in Figure 2. Our results (Table 4) showed that by increasing the HPMC and CMC concentration in gluten‐free pancakes the b* and the L* value decreased while the a* value increased. The lowest b* and L* values were for sample containing 1.5% CMC followed by 3% HPMC.

**TABLE 4:** Color values of the pancakes.

Samples	Color index			
	L* value	a* value	b* value	Δ*E*
C	47.69 ± 0.42^d^	19.96 ± 0.16^b^	53.90 ± 0.26^a^	0 ± 0.00^f^
GF	47.88 ± 0.43^d^	17.96 ± 0.09^c^	54.08 ± 0.36^a^	2.10 ± 0.12^e^
CMC 0.5	50.56 ± 0.48^c^	15.13 ± 0.07^d^	49.61 ± 0.27^b^	7.76 ± 0.06^d^
CMC 1	42.97 ± 0.49^e^	19.83 ± 0.09^b^	48.76 ± 0.18^c^	8.59 ± 0.04^c^
CMC 1.5	40.53 ± 0.24^f^	25.40 ± 0.06^a^	48.04 ± 0.22^c^	11.81 ± 0.03^a^
HPMC 1	55.13 ± 0.18^a^	13.32 ± 0.18^e^	54.23 ± 0.14^a^	10.17 ± 0.28^b^
HPMC 2	53.28 ± 0.75^b^	19.29 ± 0.08^b^	48.26 ± 0.29^c^	7.35 ± 0.17^d^
HPMC 3	41.43 ± 0.22^f^	24.71 ± 0.06^a^	48.00 ± 0.01^c^	9.94 ± 0.38^b^

*Note*: Mean ± standard deviation with different superscripts in a column differ significantly (*p* < .05) (*n* = 3). L* indicates lightness, a* is the red/green coordinate, and b* is the yellow/blue coordinate.

Abbreviations: C, control pancake (wheat flour‐based pancake); CMC, pancake containing carboxymethyl cellulose in the different levels (0.5%, 1%, and 1.5%); GF, gluten‐free pancake; HPMC, pancake containing hydroxypropyl methylcellulose in the different levels (1%, 2%, and 3%).

Color is one of the important properties of baked products. Crust color basically originates from the Maillard reaction and caramelization of sugars that occurs during baking temperatures (Mir et al., 2015). Color parameter depends on the operation condition that applies during baking such as modes of heat transfer and temperature, physicochemical characteristic of the dough like pH, relative humidity, amino acid, and reducing sugar content (Esteller & Lannes, 2008).

By addition of hydrocolloid concentration, the viscosity of pancake batter increased. Therefore, more time was needed until bubbles formed on the surface of the pancakes during the cooking. As a result, by applying more heating, the crust color of pancakes becomes darker. A similar trend was observed in gluten‐free riceberry pancakes with the addition of xanthan gum (Phongnarisorn et al., 2023).

Similar results about the darker crust of products (lower L* values) that containing HPMC have been previously reported (Kim & Yokoyama, 2011; Sabanis & Tzia, 2011). The reduction of the L* value in samples containing gum was related to the higher water‐holding capacity of gum which by maintaining moisture and cooking time caused the changes in the crust color of the final product (Moradi et al., 2021). Contrary to our results, some studies reported the increment of the L* and the b* values of the crust of cupcake containing high concentration of CMC (Abdelnaby et al., 2021).

### 3.5 Scanning electron microscopy (SEM)

**FIGURE 3:**
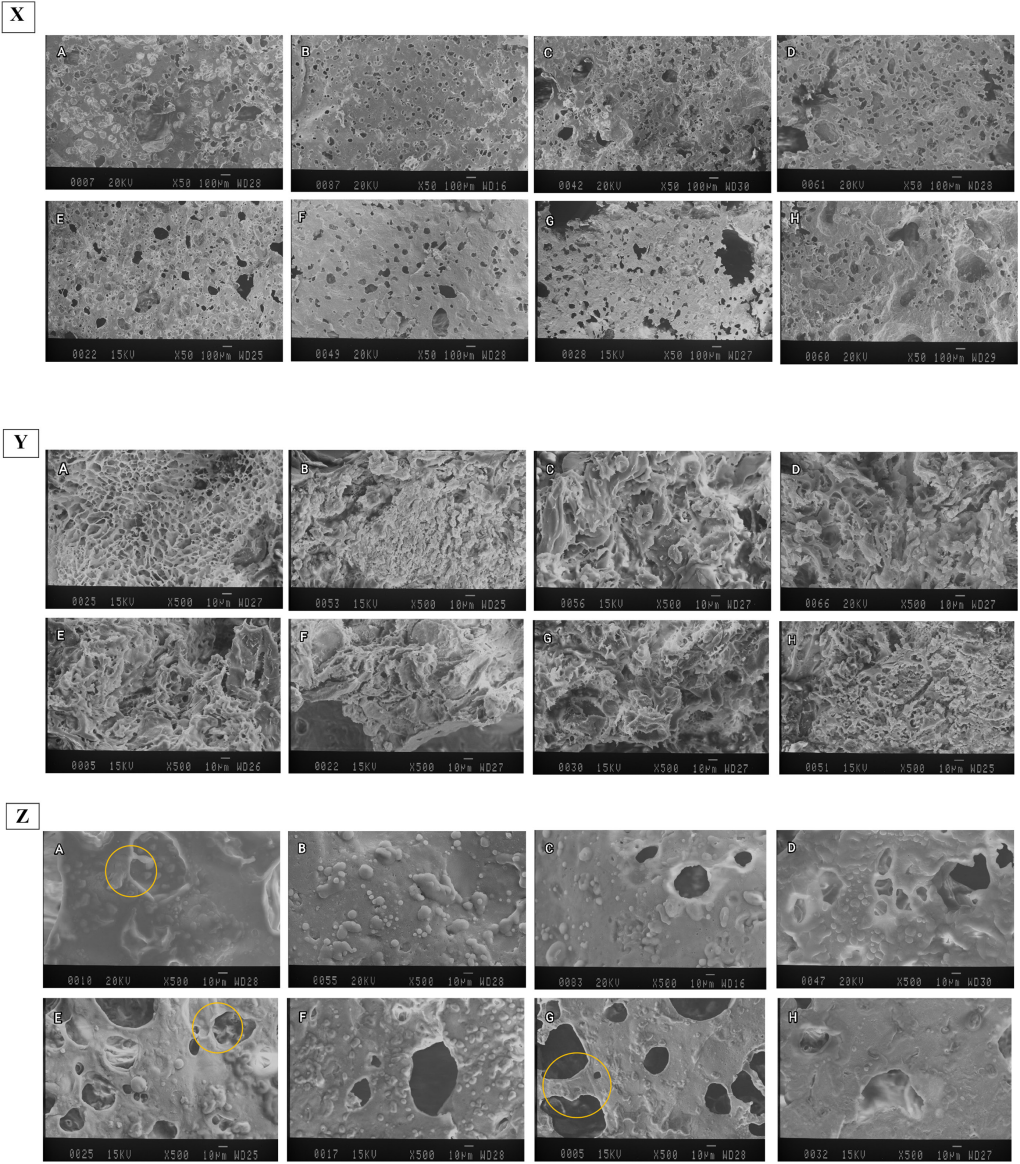
X: SEM of inner‐surface micrographs of the pancakes. 50×. Y: SEM of inner‐micrographs of the pancakes. 500×. Z: SEM of granules and gas‐cell micrographs of the pancakes. 500×. (a) wheat flour‐based sample (control); (b) gluten‐free sample; (c) gluten‐free sample containing 1% HPMC; (d) gluten‐free sample containing 2% HPMC; (e) gluten‐free sample containing 3% HPMC; (f) gluten‐free sample containing 0.5% CMC; (g) gluten‐free sample containing 1% CMC; (h) gluten‐free sample containing 1.5% CMC.

As shown in Figure 3X, the porosity observed in the inner‐surface microstructure of the pancakes was formed by air cells rupturing as the result of water migration during the cooking process. The air cells in the gluten‐free pancakes containing HPMC and CMC (Figure 3X,c–h) were larger than the gluten‐free samples without gum (Figure 3X,b). As well, the gluten‐free sample without gum had a denser structure. These porosities showed that HPMC and CMC gums could maintain more water content compared to gluten‐free and gum‐free sample, and created a similar texture to the control (wheat flour) sample. In agreement with our finding, Marco and Rosell (2008) reported that the crumb of rice bread had a dense structure and contained a large number of very small gas cells in an interrupted protein matrix. Moreover, the addition of hydrocolloids to gluten‐free doughnuts resulted in the formation of a thick layer on the air cell surface and remained a separate discrete entity of gas bubbles (Siriwongwilaichat & Kongpanichtrakul, 2021).

Contrary to our results, the addition of HPMC to gluten‐free doughnuts resulted in the formation of a denser inner structure and less porosity (Kim et al., 2015).

The inner microstructure of pancakes is indicated in Figure 3Y. The completely web‐like structure was observed in wheat flour‐based pancakes (Figure 3Y,a sample) which indicated the presence of gluten networks, while this structure was not observed in the gluten‐free sample (Figure 3Y,b sample). On the other hand, micrographs showed that HPMC and CMC help to create semi‐web‐like structures in gluten‐free pancakes. The results showed that HPMC and CMC by forming a three‐dimensional network created a structure like a control sample. In addition, the micrographs shown in Figure 3Y,c–h proved that HPMC and CMC were effective in improving the specific volume of gluten‐free pancakes. Generally, hydrocolloids were able to stabilize the gas cells in gluten‐free matrixes. In line with our findings, Salehi (2019) mentioned the ability of CMC gum to form a three‐dimensional network in the food system. Also, Ahlborn et al. (2005) mentioned that xanthan gum could create the web‐like structure in gluten‐free bread similar to wheat bread (Ahlborn et al., 2005). Also mentioned that hydrocolloids alone are not sufficient to stabilize gas cells in low‐protein starch bread. However, it was reported that the hydrocolloids in cracker samples (without the addition of proteins) were able to produce fairly stable gas cells and fairly continuous matrixes (Nammakuna et al., 2016).

The effects of hydrocolloids on starch granules and gas cells on pancakes are shown in Figure 3Z. The wheat flour sample (Figure 3Z, a) had soft semi‐smooth granules, while the gluten‐free samples (B) had different shapes of granules. According to the yellow circular marks indicated on micrographs, the samples containing HPMC (Figure 3Z,c–e) had a soft edge in walls and granules like control (wheat flour) samples (Figure 3Z,a). In contrast, pancakes containing CMC gum (Figure 3Z,f–h) had thick gas cell walls with dry and brittle edges, which can be related to its lower moisture content compared to the control (wheat flour) sample. Bárcenas and Rosell (2005) reported that the gas cell walls of the crumb of bread containing HPMC had a smoother structure with less cavities than the bread without HPMC.

### 3.6 Texture profile analysis

**FIGURE 4:**
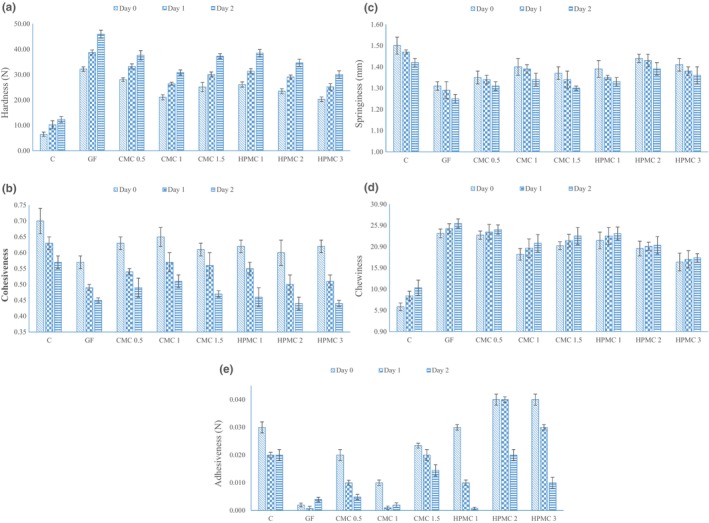
Effect of hydrocolloids on textural properties of pancake during storage time. (a) hardness; (b) cohesiveness; (c) springiness; (d) chewiness; (e) adhesiveness. C, control pancake (wheat flour‐based pancake), GF, gluten‐free pancake, CMC, pancake containing carboxymethyl cellulose in the different levels (0.5%, 1%, and 1.5%), HPMC, pancake containing hydroxypropyl methylcellulose in the different levels (1%, 2%, and 3%).

As shown in Figure 4a, the lowest and the highest hardnesses were related to the control and gluten‐free samples, respectively. These results were similar to the previous study (Siriwongwilaichat & Kongpanichtrakul, 2021). In contrast to our findings, the lower hardness was reported for gluten‐free bakery samples such as muffins and pancakes in comparison to based‐wheat flour samples (Masmoudi et al., 2020; Shih et al., 2006). HPMC and CMC had a softening effect on gluten‐free pancakes and caused hardness reduction. Several studies have reported the softening effect of hydrocolloids in different gluten‐free products (Kim & Yokoyama, 2011; Liu et al., 2018; Mohammadi et al., 2014). Gums retain water in the matrix which caused the higher moisture content of the product and consequently, retrogradation of starch and bread firming is retarded (Mohammadi et al., 2014). Our results showed the hardness of the pancakes increased during storage especially in gluten‐free samples without gum, although HPMC and CMC reduced the hardness increment rate due to their ability to weaken the starch structure (Masmoudi et al., 2020). The lower hardening rate of bakery products containing HPMC or CMC gum during storage was reported in previous studies (Kim & Yokoyama, 2011; Lazaridou et al., 2007). The effect of gums on the hardness increment during storage time depends on the type of added gums, the migration of water, and the retrogradation of starch (Itthivadhanapong et al., 2016). The suitability of gums like HPMC for the improvement of the hardness of bakery products depends on the formulation used. The gum properties are influenced by pH and ionic strength of the surrounding media as well as heat and shearing. Furthermore, the different chemical compositions of cereals may interact to different extents with gums. In conclusion, it can be said that HPMC has the potential to improve or decrease the quality properties of the flour products (Hager, 2013).

In our study, according to Figure 4b, results showed that HPMC and CMC improved the cohesiveness in gluten‐free samples compared with gluten‐free samples without gums, especially on (0) days, and first day of storage. However, the control sample had the highest cohesiveness value (0.70, 0.63, and 0.57 for storage time of 0, 1, and 2 days, respectively). Cohesiveness is defined as the force between internal links that maintain the structure of the food (Rakmai et al., 2021). The low cohesiveness caused the reduction of consumer satisfaction, because of creating crumbling. Therefore, using hydrocolloids could reduce the crumbling and increase consumer acceptance by improving this parameter (Liu et al., 2018). In previous study, higher cohesiveness value was reported for gluten‐free pancakes compared to control (wheat) samples (Shih et al., 2006). Our results indicated that the cohesiveness of all samples was decreased during storage. The same trend was reported in layer cakes (Gómez et al., 2007) and in gluten‐free bread during 5 days of storage (Moore et al., 2004). However, there was no difference in cohesiveness of gluten‐free cakes after 4 days of storage (Itthivadhanapong et al., 2016). The cohesiveness reduction during storage might related to the loss of intramolecular attraction among ingredients, drying, and the trend to crumbliness with aging (Gómez et al., 2007). Opposite results reported by Masmoudi et al. (2020). They declared that hydrocolloids did not only improve the cohesiveness value in muffins but also cause decreased it (Masmoudi et al., 2020). Also, xanthan gum had a non‐significant effect on the cohesiveness value of gluten‐free bread based on rice‐buckwheat flour in Burešová et al. (2016) study.

Based on Figure 4c, the highest and lowest springiness value during different days was related to the control (wheat flour) and the gluten‐free (without gum) samples, respectively. Springiness is defined as the ability of the sample to return to its original shape after removing the force (Rakmai et al., 2021). The lower springiness value in gluten‐free doughnuts compared with the wheat flour doughnuts has been previously reported (Kim et al., 2015). Furthermore, HPMC and CMC increased the springiness of the gluten‐free pancakes compared with the gluten‐free (without gum) sample. Similar results were found by Mohammadi et al. (2014). They reported xanthan gum improved the elastic properties of gluten‐free bread (Mohammadi et al., 2014). The ability of the gums to retain more moisture is explained as a reason for springiness improvement in bakery products (Sumnu et al., 2010). During the storage, a decreasing trend was observed for the springiness of pancake samples. A similar trend was observed during the storage of layer‐cake, gluten‐free cake, and sugar‐free cake (Azmoon et al., 2021; Gómez et al., 2007; Preichardt et al., 2011). The slight water loss during the storage day could be one of the reasons for springiness decrease (Azmoon et al., 2021). However, no significant changes regarding the impact of gums on the springiness of gluten‐free muffins and bread have been previously reported (Kittisuban et al., 2014; Masmoudi et al., 2020).

Based on Figure 4d, the control (wheat flour) sample had the lowest chewiness value, while the gluten‐free sample without gum had the highest chewiness value. Moreover, HPMC and CMC reduced the chewiness due to their softening effect. The chewiness of all pancake samples increased during storage. A similar trend was observed by Gómez et al. (2007). These authors mentioned that chewiness value is dependent on firmness, and therefore followed a similar trend of firmness changes (Gómez et al., 2007).

However, hydrocolloids are utilized to enhance the adhesive strength and improve the viscosity of batter (Masmoudi et al., 2020); our findings (Figure 4e) indicated no significant difference between the adhesiveness of samples. A similar result was found on gluten‐free pasta with different concentrations of xanthan gum (Milde et al., 2020). However, the addition of CMC gum increased the adhesiveness value in gluten‐free cakes (Mir et al., 2015).

### 3.7 Sensory evaluation

**FIGURE 5:**
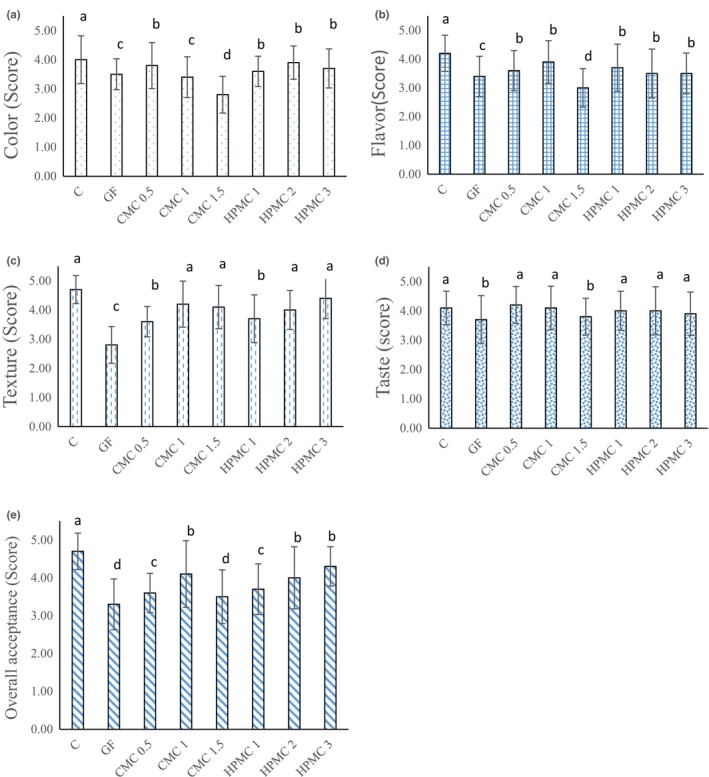
Sensory hedonic scores of pancakes: (a) color; (b) flavor; (c) texture; (d) taste; (e) overall acceptance. C, control pancake (wheat flour‐based pancake); GF, gluten‐free pancake; CMC, pancake containing carboxymethyl cellulose in the different levels (0.5%, 1%, and 1.5%); HPMC, pancake containing hydroxypropyl methylcellulose in the different levels (1%, 2%, and 3%). Lowercase letters above columns indicate the significant differences (*p* < .05).

According to Figure 5, the control (wheat flour) sample had the highest score in all sensory parameters, while the gluten‐free sample had the lowest score in most sensory parameters. The increasing trend in sensory score of taste and overall acceptance was observed with the increase of HPMC gum concentration. Our findings were similar to Siriwongwilaichat and Kongpanichtrakul (2021) study; they reported that the score of all the sensory parameters of the control doughnuts (wheat flour) was higher than the scores of all gluten‐free samples. On the other hand, the score of the sensory characteristics of the gluten‐free sample without gum was lower than samples containing gum. In addition, samples containing a higher concentration of xanthan, CMC, and HPMC gums had greater sensory scores. Also, Salehi et al. (2018) studied the effect of balangu seed gum on the sensory and physical properties of gluten‐free rice cakes. Their results showed that with increasing the concentration of gum, all sensory characteristics increased, and the lowest scores were related to the sample without gum.

In our study, gluten‐free samples containing 2 and 3% HPMC and 1% CMC had the highest score in overall acceptance, while the sample with 1.5% CMC had the lowest score of color and flavor because of the longer cooking time and high viscosity of the batter. In study done by Raungrusmee et al. (2020), noodles containing a lower concentration of xanthan showed a higher sensory score. Mir et al. (2015) declared that CMC had a special flavor and aroma which could influence on flavor and color of the crumb and the crust of the gluten‐free cake and decrease product acceptance. Therefore, it seemed that the lower scores of pancakes containing the higher concentration of CMC (1.5%) were related to the mentioned cause.

## 4 CONCLUSION

Due to the lower quality of gluten‐free products, the improvement of the textural and quality properties of these products is considered an important issue. So, in this study, gluten‐free pancakes were prepared based on rice flour and potato starch, and HPMC and CMC were used in different concentrations to improve the textural, sensory, and physicochemical properties. Results showed that the addition of both gums in gluten‐free pancakes decreased the hardness and chewiness while increasing the cohesiveness, retaining more moisture, and improving the sensory score. In addition, increasing the CMC and HPMC concentration resulted in L* value reduction, which caused a darker crust in pancakes. By increasing the HPMC gum concentrations, the viscosity and special volume of gluten‐free pancake increased significantly (*p* < .05). In general, including 3% HPMC or 1% CMC in gluten‐free pancakes could improve the quality of this product.

## AUTHOR CONTRIBUTIONS

**Maryam Maghsoud:** Data curation (equal); formal analysis (equal); methodology (equal); software (equal); validation (equal); writing – original draft (equal). **Ali Heshmati:** Conceptualization (equal); data curation (equal); formal analysis (equal); funding acquisition (equal); investigation (equal); methodology (equal); project administration (equal); resources (equal); software (equal); supervision (equal); validation (equal); visualization (equal); writing – original draft (equal); writing – review and editing (equal). **Mehdi Taheri:** Data curation (equal); investigation (equal); methodology (equal); supervision (equal). **Aryou emamifar:** Data curation (equal); formal analysis (equal); investigation (equal); project administration (equal); supervision (equal). **Fatemeh Esfarjani:** Project administration (equal); supervision (equal); validation (equal); writing – review and editing (equal).

## FUNDING INFORMATION

The author(s) received no financial support for the research, authorship, and/or publication of this article.

## CONFLICT OF INTEREST STATEMENT

The authors declare that they do not have any conflict of interest.

## ETHICS STATEMENT

The protocols and procedures utilized for this study were approved by the Research Ethics Committee of Hamadan University of Medical Sciences, Hamadan, Iran (Ethical code: IR.UMSHA.REC.1401.792).

## CONSENT TO PUBLISH

The authors declare their consent to publish this article.

## Data Availability

The data that support the findings of this study are available from the corresponding author, upon reasonable request.
